# *tert*-Butyl 3-[*N*-(*tert*-butoxy­carbonyl)methyl­amino]-4-methoxy­imino-3-methyl­piperidine-1-carboxyl­ate

**DOI:** 10.1107/S1600536808044255

**Published:** 2009-01-08

**Authors:** Zhilong Wan, Yun Chai, Mingliang Liu, Huiyuan Guo

**Affiliations:** a Institute of Medicinal Biotechnology, Chinese Academy of Medical Sciences and Peking Union Medical College, Beijing 100050, People’s Republic of China

## Abstract

The title compound, C_18_H_33_N_3_O_5_, was prepared from *N*-*tert*-butoxy­carbonyl-4-piperidone using a nine-step reaction, including condensation, methyl­ation, oximation, hydrolysis, esterification, ammonolysis, Hoffmann degradation, *tert*-butoxy­carbonyl protection and methyl­ation. The *E* configuration of the methyl­oxime geometry of the compound is confirmed.

## Related literature

For the synthesis and properties of quinolone derivatives, see: Anderson & Osheroff (2001 ▶); Ball *et al.* (1998 ▶); Hong *et al.* (1997 ▶); Ray *et al.* (2005 ▶); Wang *et al.* (2008 ▶).
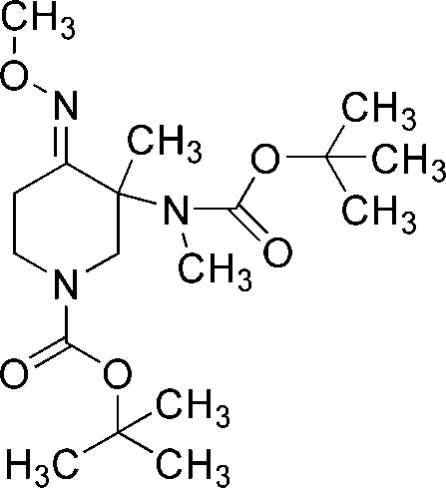


## Experimental

### 

#### Crystal data

C_18_H_33_N_3_O_5_
*M* *_r_* = 371.47
Monoclinic, 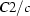

*a* = 28.867 (3) Å
*b* = 6.1887 (13) Å
*c* = 25.379 (3) Å
β = 112.769 (2)°
*V* = 4180.6 (11) Å^3^
*Z* = 8
Mo *K*α radiation
μ = 0.09 mm^−1^
*T* = 298 (2) K
0.40 × 0.20 × 0.11 mm

#### Data collection

Bruker SMART CCD area-detector diffractometer
Absorption correction: multi-scan (*SADABS*; Sheldrick, 1996 ▶) *T*_min_ = 0.963, *T*_max_ = 0.991
10032 measured reflections
3699 independent reflections
1915 reflections with *I* > 2σ(*I*)
*R*_int_ = 0.060

#### Refinement

*R*[*F* ^2^ > 2σ(*F* ^2^)] = 0.050
*wR*(*F* ^2^) = 0.144
*S* = 1.02
3699 reflections
244 parameters
H-atom parameters constrained
Δρ_max_ = 0.23 e Å^−3^
Δρ_min_ = −0.22 e Å^−3^

### 

Data collection: *SMART* (Bruker, 1998 ▶); cell refinement: *SAINT* (Bruker, 1999 ▶); data reduction: *SAINT*; program(s) used to solve structure: *SHELXS97* (Sheldrick, 2008 ▶); program(s) used to refine structure: *SHELXL97* (Sheldrick, 2008 ▶); molecular graphics: *SHELXTL* (Sheldrick, 2008 ▶); software used to prepare material for publication: *SHELXTL*.

## Supplementary Material

Crystal structure: contains datablocks global, I. DOI: 10.1107/S1600536808044255/rk2125sup1.cif

Structure factors: contains datablocks I. DOI: 10.1107/S1600536808044255/rk2125Isup2.hkl

Additional supplementary materials:  crystallographic information; 3D view; checkCIF report

## Figures and Tables

**Fig. 1.**
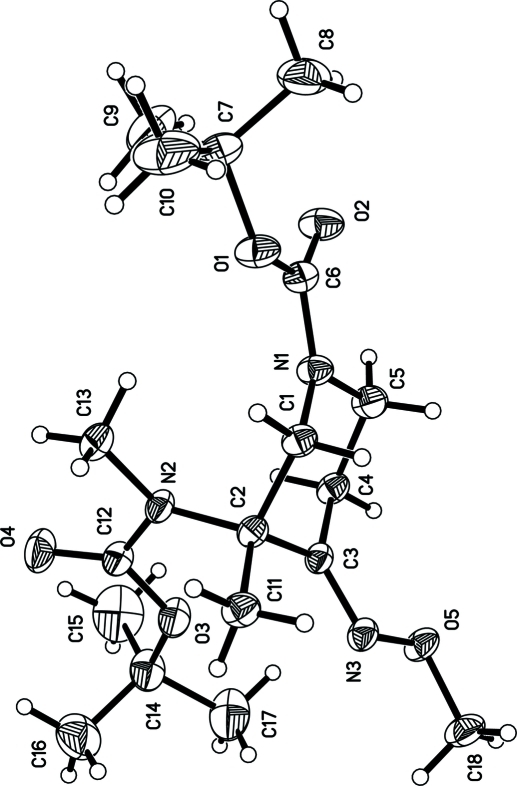
The molecular structure of the title compound with the atom–numbering scheme. Displacement ellipsoids are drawn at 40% probability level. H atoms are presented as a small spheres of arbitrary radius.

## supplementary crystallographic information

### Comment

Quinolones, a class of synthetic antibacterial compounds based on a 4–quinolone skeleton, have been the landmark discovery in the treatment of bacterial infections in both community and hospital setting (Ray *et al.*, 2005; Ball *et al.*, 1998;). The most intensive structural variations have been carried out on the basic group at the C–7 position, partially due to the ease of their introduction through a nucleophilic aromatic substitution reaction on the corresponding halide. Piperazine, piperidine, pyrrolidine and their derivatives have been the most successfully employed side chains, as evidenced by the compounds currently on the market (Anderson & Osheroff, 2001; Hong *et al.*, 1997). Recently, as part of an ongoing program to find potent new quinolones displaying strong Gram–positive activity, we have focused our attention on introducing new functional groups to the piperidine ring (Wang *et al.*, 2008). We report here the crystal structure of the title compound, which is a key intermediate of 4–methoxyimino–3–methylamino–3–methylpiperidine, a novel C–7 substituent of the quinolones.

The oxime geometry of the title compound was confirmed to have the *E*–configuration. In the molecule of the compound (Fig. 1), the N1—C6 (1.352 (3) Å) and N2—C12 (1.359 (3) Å) bond lengths are significantly shorter than the normal C—N bond (1.47 Å), indicating some conjugation with the C6═O2 and C12═O4 carbonyl groups, respectively. The six–membered piperidine ring adopts a chair conformation with displacing N1 and C3 atoms (0.593 (3) Å and -0.654 (3) Å respectively) from the mean–plane (C1, C2, C4 and C5).

### Experimental

To a stirring solution of 1-*N*-*tert*-Butoxycarbonyl-3-(*N*-*tert*-butoxycarbonyl) amino-4-methoxyimino-3-methylpiperidine(2.4 g, 6.7 mmol) in dry tetrahydrofuran (40 ml) was added 70% sodium hydride (0.46 g, 13.4 mmol) at 273 K using an ice bath, and then stirred for 0.5 h at the room temperature. After addition of methyl iodide (0.84 ml, 13.4 mol), the reaction mixture was stirred at 313 K for 5 h and cooled to room temperature, adjusted to pH 7 with 1*N* HCl and then concentrated under reduced pressure. The residue was diluted with ethyl acetate (50 ml), washed with distilled water, dried over anhydrous sodium sulfate, and filtered. The filtrate was concentrated under reduced pressure, dried *in vacuo* to give the title compound as a white solid (2.37 g, 95.0%; mp: 380–382 K). Single crystals suitable for X–ray analysis were obtained by slow evaporation of a methanol/water solution (5:1 *v*/*v*). ^1^H NMR (CDCl_3_, δ): 1.34 (s, 3H, CH_3_), 1.41 (s, 9H, *BOC*), 1.46 (s, 9H, *BOC*), 2.24–2.25 (m, 1H, piperidine), 2.87–2.88 (m, 1H, piperidine), 2.91 (s, 3H, NCH_3_), 2.95–3.08 (m, 2H, piperidine), 3.82 (s, 3H, OCH_3_), 3.84–3.86 (m, 1H, piperidine), 4.30–4.31 (m, 1H, piperidine); MS (ESI, m/*z*): 372.2 m/*z* (*M*+1)^+^.

### Refinement

All H atoms were placed at calculated positions, with C—H = 0.96–0.97 Å, and included in the final cycles of refinement using a riding model, with *U*_iso_(H) = 1.2*U*_eq_(C) for methylene or 1.5*U*_eq_(C) for methyl H atoms.

### Crystal data

**Table:**

C_18_H_33_N_3_O_5_	*F*(000) = 1616
*M**_r_* = 371.47	*D*_x_ = 1.180 Mg m^−^^3^
Monoclinic, *C*2/*c*	Mo *K*α radiation, λ = 0.71073 Å
Hall symbol: -C 2yc	Cell parameters from 1707 reflections
*a* = 28.867 (3) Å	θ = 2.7–21.1°
*b* = 6.1887 (13) Å	µ = 0.09 mm^−^^1^
*c* = 25.379 (3) Å	*T* = 298 K
β = 112.769 (2)°	Prism, colourless
*V* = 4180.6 (11) Å^3^	0.40 × 0.20 × 0.11 mm
*Z* = 8	

### Data collection

**Table:**

Bruker SMART CCD area-detector diffractometer	3699 independent reflections
Radiation source: Fine–focus sealed tube	1915 reflections with *I* > 2σ(*I*)
Graphite	*R*_int_ = 0.060
φ and ω scans	θ_max_ = 25.0°, θ_min_ = 1.5°
Absorption correction: multi-scan (*SADABS*; Sheldrick, 1996)	*h* = −33→34
*T*_min_ = 0.963, *T*_max_ = 0.991	*k* = −7→6
10032 measured reflections	*l* = −30→23

### Refinement

**Table:**

Refinement on *F*^2^	Primary atom site location: structure-invariant direct methods
Least-squares matrix: full	Secondary atom site location: difference Fourier map
*R*[*F*^2^ > 2σ(*F*^2^)] = 0.050	Hydrogen site location: inferred from neighbouring sites
*wR*(*F*^2^) = 0.144	H-atom parameters constrained
*S* = 1.02	*w* = 1/[σ^2^(*F*_o_^2^) + (0.058*P*)^2^ + 0.3657*P*] where *P* = (*F*_o_^2^ + 2*F*_c_^2^)/3
3699 reflections	(Δ/σ)_max_ = 0.001
244 parameters	Δρ_max_ = 0.23 e Å^−^^3^
0 restraints	Δρ_min_ = −0.22 e Å^−^^3^

### Special details

**Table:**

Geometry. All s.u.'s (except the s.u. in the dihedral angle between two l.s. planes) are estimated using the full covariance matrix. The cell s.u.'s are taken into account individually in the estimation of s.u.'s in distances, angles and torsion angles; correlations between s.u.'s in cell parameters are only used when they are defined by crystal symmetry. An approximate (isotropic) treatment of cell s.u.'s is used for estimating s.u.'s involving l.s. planes.
Refinement. Refinement of *F*^2^ against ALL reflections. The weighted *R*–factor *wR* and goodness of fit *S* are based on *F*^2^, conventional *R*–factors *R* are based on *F*, with *F* set to zero for negative *F*^2^. The threshold expression of *F*^2^ > σ(*F*^2^) is used only for calculating *R*–factors(gt) *etc*. and is not relevant to the choice of reflections for refinement. *R*–factors based on *F*^2^ are statistically about twice as large as those based on *F*, and *R*–factors based on ALL data will be even larger.

### Fractional atomic coordinates and isotropic or equivalent isotropic displacement parameters (Å^2^)

**Table:**

	*x*	*y*	*z*	*U*_iso_*/*U*_eq_	
N1	0.18007 (8)	0.2543 (3)	0.60235 (9)	0.0389 (6)	
N2	0.07900 (7)	0.1687 (4)	0.51326 (9)	0.0367 (6)	
N3	0.16321 (7)	−0.1321 (4)	0.47432 (9)	0.0382 (6)	
O1	0.16324 (7)	0.3113 (3)	0.67977 (7)	0.0496 (5)	
O2	0.21149 (7)	0.5507 (3)	0.65653 (8)	0.0554 (6)	
O3	0.07112 (6)	0.1423 (3)	0.42229 (7)	0.0525 (6)	
O4	0.01093 (7)	0.3134 (3)	0.44214 (8)	0.0607 (6)	
O5	0.19807 (6)	−0.0836 (3)	0.44880 (7)	0.0452 (5)	
C1	0.15544 (9)	0.0442 (4)	0.59370 (10)	0.0374 (7)	
H1A	0.1361	0.0344	0.6175	0.045*	
H1B	0.1809	−0.0681	0.6057	0.045*	
C2	0.12010 (9)	0.0043 (4)	0.53070 (10)	0.0335 (6)	
C3	0.15423 (9)	0.0354 (4)	0.49846 (10)	0.0325 (6)	
C4	0.17891 (10)	0.2510 (4)	0.50509 (11)	0.0387 (7)	
H4A	0.1536	0.3625	0.4899	0.046*	
H4B	0.2006	0.2542	0.4839	0.046*	
C5	0.20975 (10)	0.2938 (5)	0.56825 (11)	0.0459 (8)	
H5A	0.2390	0.2005	0.5813	0.055*	
H5B	0.2213	0.4425	0.5732	0.055*	
C6	0.18713 (10)	0.3856 (5)	0.64739 (11)	0.0399 (7)	
C7	0.15940 (11)	0.4430 (5)	0.72637 (12)	0.0495 (8)	
C8	0.21026 (13)	0.4764 (6)	0.77309 (13)	0.0738 (11)	
H8A	0.2261	0.3388	0.7856	0.111*	
H8B	0.2066	0.5494	0.8046	0.111*	
H8C	0.2306	0.5623	0.7589	0.111*	
C9	0.13357 (14)	0.6544 (6)	0.70277 (15)	0.0871 (13)	
H9A	0.1561	0.7461	0.6934	0.131*	
H9B	0.1241	0.7241	0.7309	0.131*	
H9C	0.1041	0.6272	0.6690	0.131*	
C10	0.12728 (14)	0.3024 (6)	0.74676 (14)	0.0903 (13)	
H10A	0.0957	0.2762	0.7158	0.135*	
H10B	0.1216	0.3738	0.7773	0.135*	
H10C	0.1441	0.1674	0.7603	0.135*	
C11	0.09808 (10)	−0.2224 (4)	0.52538 (12)	0.0432 (7)	
H11A	0.0769	−0.2306	0.5466	0.065*	
H11B	0.1248	−0.3256	0.5403	0.065*	
H11C	0.0786	−0.2537	0.4859	0.065*	
C12	0.05026 (10)	0.2153 (5)	0.45784 (12)	0.0417 (7)	
C13	0.05602 (10)	0.2229 (5)	0.55381 (11)	0.0492 (8)	
H13A	0.0295	0.3257	0.5367	0.074*	
H13B	0.0810	0.2841	0.5877	0.074*	
H13C	0.0425	0.0944	0.5636	0.074*	
C14	0.04913 (11)	0.1885 (6)	0.36115 (12)	0.0584 (9)	
C15	0.04440 (16)	0.4286 (7)	0.35091 (17)	0.1048 (14)	
H15A	0.0185	0.4844	0.3620	0.157*	
H15B	0.0360	0.4573	0.3111	0.157*	
H15C	0.0757	0.4972	0.3731	0.157*	
C17	0.08735 (13)	0.0915 (7)	0.34093 (13)	0.0907 (13)	
H17A	0.1198	0.1530	0.3622	0.136*	
H17B	0.0779	0.1217	0.3010	0.136*	
H17C	0.0887	−0.0621	0.3467	0.136*	
C16	−0.00059 (12)	0.0715 (7)	0.33491 (14)	0.0906 (13)	
H16A	0.0039	−0.0778	0.3460	0.136*	
H16B	−0.0129	0.0822	0.2940	0.136*	
H16C	−0.0244	0.1360	0.3481	0.136*	
C18	0.20473 (11)	−0.2741 (5)	0.42114 (13)	0.0557 (9)	
H18A	0.2217	−0.3815	0.4493	0.083*	
H18B	0.2245	−0.2411	0.3992	0.083*	
H18C	0.1725	−0.3283	0.3962	0.083*	

### Atomic displacement parameters (Å^2^)

**Table:**

	*U*^11^	*U*^22^	*U*^33^	*U*^12^	*U*^13^	*U*^23^
N1	0.0452 (13)	0.0367 (15)	0.0395 (13)	−0.0085 (11)	0.0215 (11)	−0.0060 (12)
N2	0.0359 (12)	0.0426 (15)	0.0368 (13)	0.0076 (11)	0.0198 (11)	0.0002 (11)
N3	0.0368 (12)	0.0427 (15)	0.0420 (13)	0.0011 (11)	0.0226 (11)	−0.0024 (12)
O1	0.0665 (13)	0.0501 (13)	0.0394 (11)	−0.0098 (11)	0.0284 (10)	−0.0081 (10)
O2	0.0687 (14)	0.0486 (14)	0.0528 (13)	−0.0193 (12)	0.0277 (11)	−0.0143 (11)
O3	0.0471 (11)	0.0771 (16)	0.0335 (11)	0.0188 (11)	0.0157 (9)	0.0062 (11)
O4	0.0469 (12)	0.0716 (16)	0.0619 (14)	0.0252 (12)	0.0193 (10)	0.0080 (12)
O5	0.0509 (11)	0.0453 (13)	0.0538 (12)	−0.0003 (10)	0.0360 (10)	−0.0070 (10)
C1	0.0438 (16)	0.0357 (18)	0.0371 (16)	−0.0012 (14)	0.0203 (13)	−0.0006 (13)
C2	0.0361 (15)	0.0331 (17)	0.0349 (15)	0.0017 (13)	0.0178 (12)	0.0011 (13)
C3	0.0314 (14)	0.0359 (17)	0.0331 (15)	0.0034 (13)	0.0155 (12)	0.0006 (13)
C4	0.0451 (16)	0.0350 (17)	0.0442 (17)	−0.0010 (14)	0.0264 (13)	−0.0012 (14)
C5	0.0468 (17)	0.0457 (19)	0.0510 (18)	−0.0088 (15)	0.0254 (15)	−0.0077 (15)
C6	0.0454 (17)	0.0398 (19)	0.0361 (16)	0.0002 (15)	0.0174 (14)	−0.0027 (15)
C7	0.066 (2)	0.050 (2)	0.0392 (17)	0.0003 (17)	0.0280 (16)	−0.0052 (16)
C8	0.090 (3)	0.084 (3)	0.0401 (18)	0.001 (2)	0.0172 (19)	−0.0105 (19)
C9	0.110 (3)	0.089 (3)	0.074 (3)	0.044 (3)	0.049 (2)	0.007 (2)
C10	0.127 (3)	0.099 (3)	0.071 (2)	−0.034 (3)	0.066 (2)	−0.023 (2)
C11	0.0486 (16)	0.0375 (18)	0.0494 (17)	−0.0058 (14)	0.0252 (14)	−0.0037 (15)
C12	0.0406 (17)	0.0440 (19)	0.0439 (18)	0.0051 (15)	0.0201 (14)	0.0005 (15)
C13	0.0461 (17)	0.058 (2)	0.0512 (18)	0.0062 (15)	0.0276 (15)	−0.0066 (16)
C14	0.054 (2)	0.082 (3)	0.0373 (17)	0.0154 (19)	0.0156 (15)	0.0125 (18)
C15	0.120 (3)	0.109 (4)	0.092 (3)	0.012 (3)	0.048 (3)	0.044 (3)
C17	0.079 (2)	0.151 (4)	0.046 (2)	0.030 (3)	0.0297 (19)	0.010 (2)
C16	0.072 (3)	0.130 (4)	0.057 (2)	0.002 (3)	0.0116 (19)	−0.011 (2)
C18	0.065 (2)	0.052 (2)	0.065 (2)	−0.0031 (17)	0.0409 (17)	−0.0190 (17)

### Geometric parameters (Å, °)

**Table:**

N1—C6	1.352 (3)	C8—H8B	0.9600
N1—C5	1.455 (3)	C8—H8C	0.9600
N1—C1	1.457 (3)	C9—H9A	0.9600
N2—C12	1.359 (3)	C9—H9B	0.9600
N2—C13	1.463 (3)	C9—H9C	0.9600
N2—C2	1.494 (3)	C10—H10A	0.9600
N3—C3	1.280 (3)	C10—H10B	0.9600
N3—O5	1.423 (2)	C10—H10C	0.9600
O1—C6	1.342 (3)	C11—H11A	0.9600
O1—C7	1.476 (3)	C11—H11B	0.9600
O2—C6	1.210 (3)	C11—H11C	0.9600
O3—C12	1.342 (3)	C13—H13A	0.9600
O3—C14	1.459 (3)	C13—H13B	0.9600
O4—C12	1.211 (3)	C13—H13C	0.9600
O5—C18	1.423 (3)	C14—C15	1.505 (5)
C1—C2	1.548 (3)	C14—C17	1.510 (4)
C1—H1A	0.9700	C14—C16	1.513 (4)
C1—H1B	0.9700	C15—H15A	0.9600
C2—C3	1.517 (3)	C15—H15B	0.9600
C2—C11	1.525 (3)	C15—H15C	0.9600
C3—C4	1.491 (3)	C17—H17A	0.9600
C4—C5	1.525 (3)	C17—H17B	0.9600
C4—H4A	0.9700	C17—H17C	0.9600
C4—H4B	0.9700	C16—H16A	0.9600
C5—H5A	0.9700	C16—H16B	0.9600
C5—H5B	0.9700	C16—H16C	0.9600
C7—C8	1.502 (4)	C18—H18A	0.9600
C7—C10	1.502 (4)	C18—H18B	0.9600
C7—C9	1.510 (4)	C18—H18C	0.9600
C8—H8A	0.9600		
			
C6—N1—C5	118.1 (2)	C7—C9—H9C	109.5
C6—N1—C1	124.7 (2)	H9A—C9—H9C	109.5
C5—N1—C1	115.2 (2)	H9B—C9—H9C	109.5
C12—N2—C13	114.7 (2)	C7—C10—H10A	109.5
C12—N2—C2	123.2 (2)	C7—C10—H10B	109.5
C13—N2—C2	118.2 (2)	H10A—C10—H10B	109.5
C3—N3—O5	110.9 (2)	C7—C10—H10C	109.5
C6—O1—C7	121.1 (2)	H10A—C10—H10C	109.5
C12—O3—C14	121.7 (2)	H10B—C10—H10C	109.5
C18—O5—N3	107.7 (2)	C2—C11—H11A	109.5
N1—C1—C2	112.7 (2)	C2—C11—H11B	109.5
N1—C1—H1A	109.1	H11A—C11—H11B	109.5
C2—C1—H1A	109.1	C2—C11—H11C	109.5
N1—C1—H1B	109.1	H11A—C11—H11C	109.5
C2—C1—H1B	109.1	H11B—C11—H11C	109.5
H1A—C1—H1B	107.8	O4—C12—O3	123.7 (3)
N2—C2—C3	111.2 (2)	O4—C12—N2	124.5 (3)
N2—C2—C11	110.1 (2)	O3—C12—N2	111.8 (2)
C3—C2—C11	113.9 (2)	N2—C13—H13A	109.5
N2—C2—C1	109.1 (2)	N2—C13—H13B	109.5
C3—C2—C1	103.36 (19)	H13A—C13—H13B	109.5
C11—C2—C1	108.9 (2)	N2—C13—H13C	109.5
N3—C3—C4	127.0 (2)	H13A—C13—H13C	109.5
N3—C3—C2	116.7 (2)	H13B—C13—H13C	109.5
C4—C3—C2	115.7 (2)	O3—C14—C15	110.5 (3)
C3—C4—C5	109.4 (2)	O3—C14—C17	102.1 (2)
C3—C4—H4A	109.8	C15—C14—C17	111.3 (3)
C5—C4—H4A	109.8	O3—C14—C16	108.8 (3)
C3—C4—H4B	109.8	C15—C14—C16	112.9 (3)
C5—C4—H4B	109.8	C17—C14—C16	110.7 (3)
H4A—C4—H4B	108.2	C14—C15—H15A	109.5
N1—C5—C4	110.9 (2)	C14—C15—H15B	109.5
N1—C5—H5A	109.5	H15A—C15—H15B	109.5
C4—C5—H5A	109.5	C14—C15—H15C	109.5
N1—C5—H5B	109.5	H15A—C15—H15C	109.5
C4—C5—H5B	109.5	H15B—C15—H15C	109.5
H5A—C5—H5B	108.0	C14—C17—H17A	109.5
O2—C6—O1	124.7 (3)	C14—C17—H17B	109.5
O2—C6—N1	123.8 (3)	H17A—C17—H17B	109.5
O1—C6—N1	111.5 (3)	C14—C17—H17C	109.5
O1—C7—C8	110.8 (2)	H17A—C17—H17C	109.5
O1—C7—C10	101.8 (2)	H17B—C17—H17C	109.5
C8—C7—C10	110.7 (3)	C14—C16—H16A	109.5
O1—C7—C9	109.8 (2)	C14—C16—H16B	109.5
C8—C7—C9	112.1 (3)	H16A—C16—H16B	109.5
C10—C7—C9	111.3 (3)	C14—C16—H16C	109.5
C7—C8—H8A	109.5	H16A—C16—H16C	109.5
C7—C8—H8B	109.5	H16B—C16—H16C	109.5
H8A—C8—H8B	109.5	O5—C18—H18A	109.5
C7—C8—H8C	109.5	O5—C18—H18B	109.5
H8A—C8—H8C	109.5	H18A—C18—H18B	109.5
H8B—C8—H8C	109.5	O5—C18—H18C	109.5
C7—C9—H9A	109.5	H18A—C18—H18C	109.5
C7—C9—H9B	109.5	H18B—C18—H18C	109.5
H9A—C9—H9B	109.5		
			
C3—N3—O5—C18	−177.9 (2)	C6—N1—C5—C4	−143.2 (2)
C6—N1—C1—C2	139.0 (2)	C1—N1—C5—C4	52.3 (3)
C5—N1—C1—C2	−57.6 (3)	C3—C4—C5—N1	−49.9 (3)
C12—N2—C2—C3	48.3 (3)	C7—O1—C6—O2	8.0 (4)
C13—N2—C2—C3	−155.2 (2)	C7—O1—C6—N1	−171.1 (2)
C12—N2—C2—C11	−78.9 (3)	C5—N1—C6—O2	10.3 (4)
C13—N2—C2—C11	77.7 (3)	C1—N1—C6—O2	173.3 (3)
C12—N2—C2—C1	161.6 (2)	C5—N1—C6—O1	−170.6 (2)
C13—N2—C2—C1	−41.8 (3)	C1—N1—C6—O1	−7.6 (4)
N1—C1—C2—N2	−62.4 (3)	C6—O1—C7—C8	−66.1 (3)
N1—C1—C2—C3	56.0 (3)	C6—O1—C7—C10	176.2 (3)
N1—C1—C2—C11	177.4 (2)	C6—O1—C7—C9	58.3 (3)
O5—N3—C3—C4	−5.2 (3)	C14—O3—C12—O4	4.0 (4)
O5—N3—C3—C2	−176.31 (19)	C14—O3—C12—N2	−175.0 (2)
N2—C2—C3—N3	−129.7 (2)	C13—N2—C12—O4	7.3 (4)
C11—C2—C3—N3	−4.6 (3)	C2—N2—C12—O4	164.6 (3)
C1—C2—C3—N3	113.4 (2)	C13—N2—C12—O3	−173.7 (2)
N2—C2—C3—C4	58.2 (3)	C2—N2—C12—O3	−16.5 (4)
C11—C2—C3—C4	−176.7 (2)	C12—O3—C14—C15	57.0 (4)
C1—C2—C3—C4	−58.8 (3)	C12—O3—C14—C17	175.5 (3)
N3—C3—C4—C5	−113.4 (3)	C12—O3—C14—C16	−67.5 (4)
C2—C3—C4—C5	57.8 (3)		

## References

[bb1] Anderson, V. E. & Osheroff, N. (2001). *Curr. Pharm. Des.* **7**, 337–353. 10.2174/1381612013398013 11254893

[bb2] Ball, P., Tilloston, G. & Fernald, A. (1998). *Expert Opin. Investig. Drugs*, **7**, 761–783. 10.1517/13543784.7.5.761 15991967

[bb3] Bruker (1998). *SMART* Bruker AXS Inc., Madison, Wisconsin, USA.

[bb4] Bruker (1999). *SAINT* Bruker AXS Inc., Madison, Wisconsin, USA.

[bb5] Hong, C. Y., Kim, Y. K. & Chang, J. H. (1997). *J. Med. Chem.* **40**, 3584–3593. 10.1021/jm970202e 9357525

[bb6] Ray, S., Pathak, S. R. & Chaturvedi, D. (2005). *Drugs Future*, **30**, 161–180.

[bb7] Sheldrick, G. M. (1996). *SADABS* University of Göttingen, Germany.

[bb8] Sheldrick, G. M. (2008). *Acta Cryst.* A**64**, 112–122. 10.1107/S0108767307043930 18156677

[bb9] Wang, X. Y., Guo, Q. & Wang, Y. C. (2008). *Acta Pharmacol. Sin.* **43**, 819–827.

